# Supplementary material for: Genome-wide analysis of DNA-PK-bound MRN cleavage products supports a sequential model of DSB repair pathway choice
Source: Nat Commun. 2023 Sep 16;14:5759. doi: 10.1038/s41467-023-41544-8 (PMC10505227; doi:10.1038/s41467-023-41544-8)
Supplement: Supplementary file 1 — Supplementary Information [file 41467_2023_41544_MOESM1_ESM.pdf]

Supplemental Figures

Figure S1. Examples of GLASS-ChIP sites where phospho-DNA-PKcs is bound in the human genome.

Figure S2. Examples of genome browser views of non-AsiSI sites where DNA-PK is bound in the human genome.

Figure S3. Analysis of ChIP-seq reads spanning the center of AsiSI and Cas9 cut sites.

Figure S4. DNA-PKcs released fragments and pellet-ChIP efficiency correlate with DSB markers.

Figure S5. Resection at AsiSI breaks is observed in all cell cycle phases.

Figure S6. A subset of DNA-PKcs binding sites coincide with other DSB markers.

Figure S7. Mass Spectrometry identifies proteins associated with released DNA-PKcs GLASS-ChIP fragments.

Figure S8. qPCR primers used for resection and for ChIP-qPCR

Supplementary Data 1. proteins identified by mass spectrometry from GLASS-ChIP immunoprecipitations.

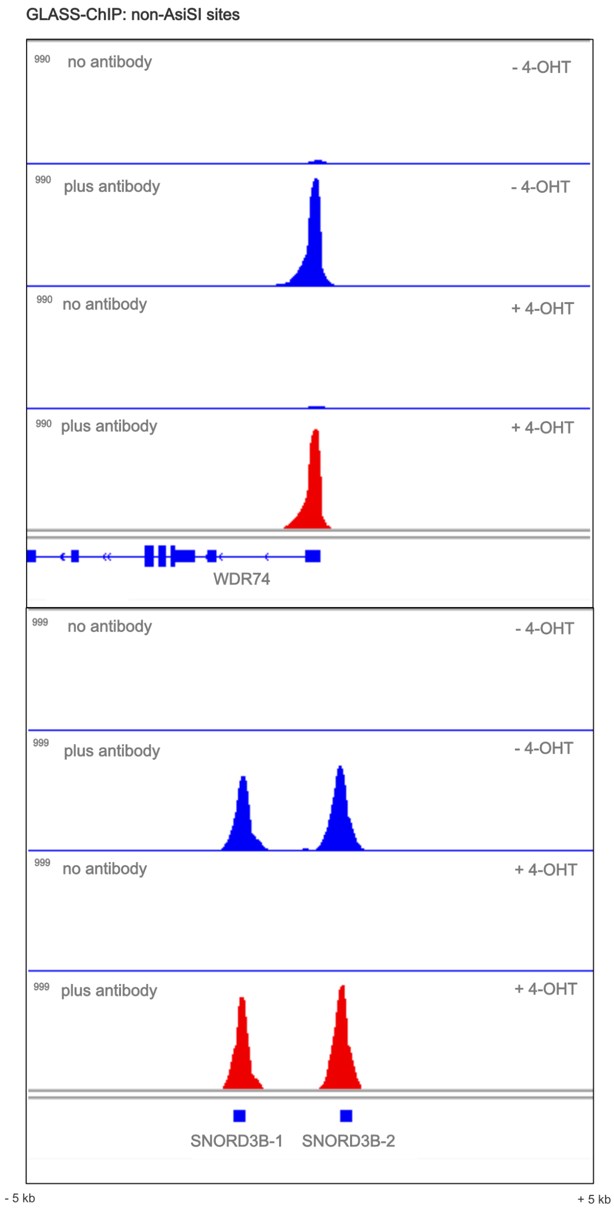

Figure S1. Examples of GLASS-ChIP sites where phospho-DNA-PKcs is bound in the human genome. No-antibody or Plus-antibody GLASS-ChIP library signals in the absence or presence of 4-OHT are shown as indicated.

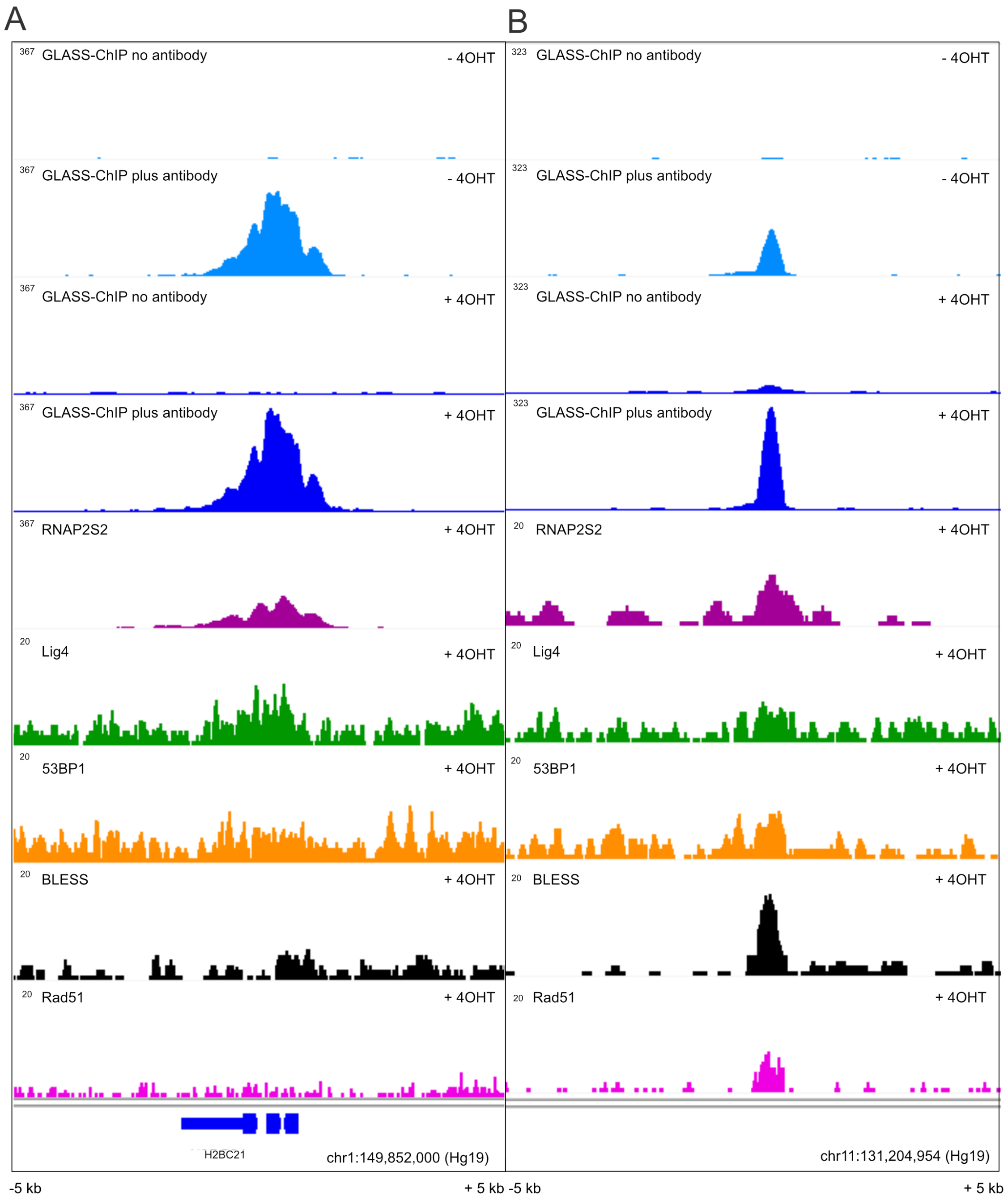

Figure S2. Examples of genome browser views of non-AsiSI sites where DNA-PK is bound in the human genome. (A,B) GLASS-ChIP (- and + 4-OHT), RNA Pol II phospho-S2 (RNAP2S2), Ligase 4, 53BP1, BLESS (DSBs), and Rad51 from this study as well as previous studies[1,2].

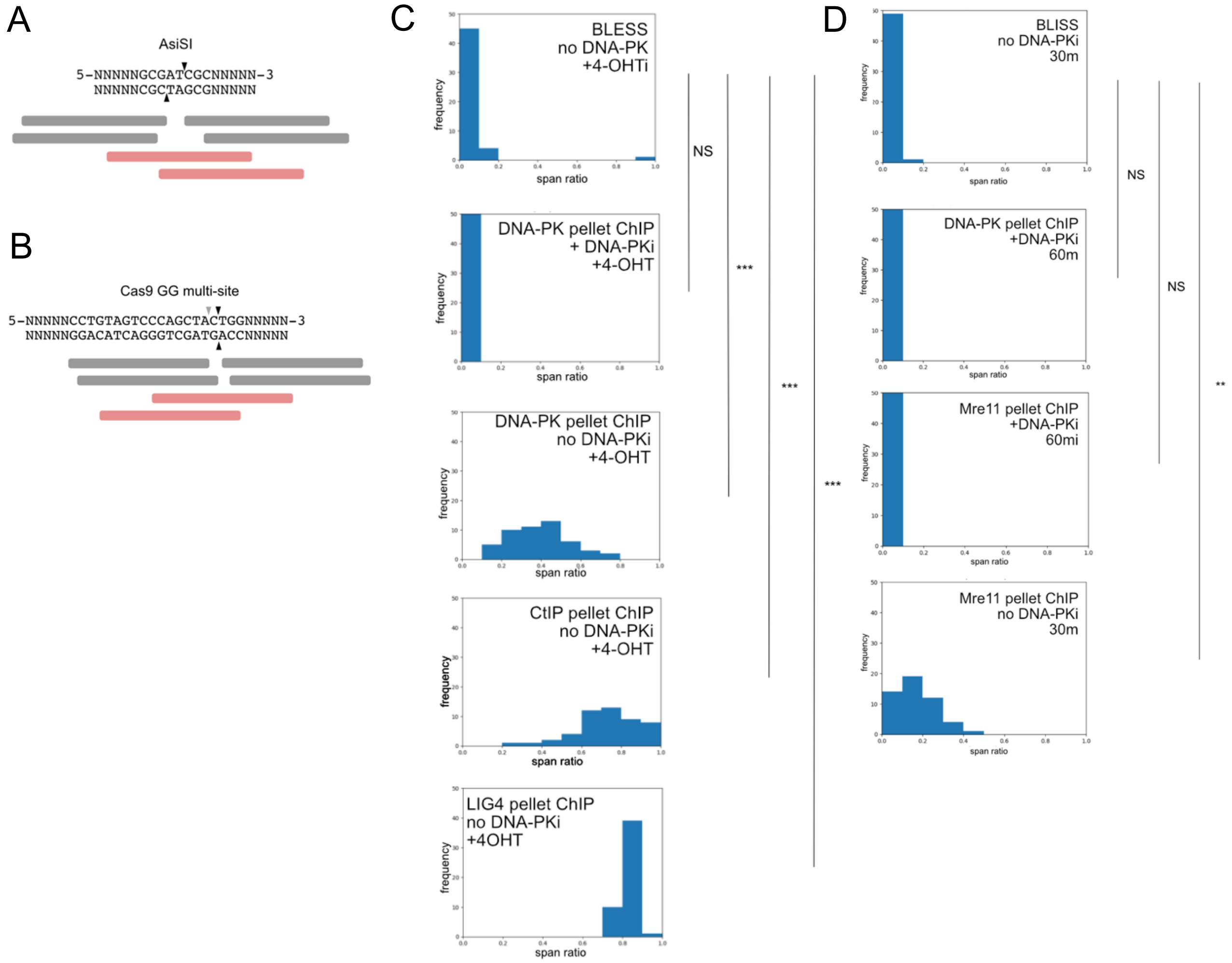

Figure S3. Analysis of ChIP-seq reads spanning the center of AsiSI and Cas9 cut sites. (A) Diagram of AsiSI recognition site and cut locations with examples of reads that do not span the cut site (grey) and reads that do span the cut site (pink). DNA fragments containing the 2 nt 3´ overhang generated by AsiSI would be removed by treatment with the end repair enzyme mix (NEB) during library preparation, thus generating a gap in processed reads for proteins bound to an unrepaired, AsiSI-cut end. (B) Diagram of Cas9 GG multi-site target with reads shown as in (A). Cas9 generates blunt ends (black) but can also generate a staggered cut as shown (grey). (C) Read "span ratios" at the 50 top AsiSI cut sites for each respective ChIP experiment. The 50 top AsiSI cut sites analyzed here are the 50 cut sites that had the highest total read count across all ChIP experiments. Span ratio is defined as the ratio of the number of reads that span an AsiSI cut site divided by the total number of reads that fall within 5nt upstream or downstream of a cut site. Pairwise Kolmogorov Smirnov statistics were determined with 1,000 bootstraps for each pairwise comparison listed. (D) Read "span ratios" for the top 50 Cas9 target sites analyzed for BLISS, DNA-PK pellet-ChIP, Mre11 pellet-ChIP with DNA-PKi, and Mre11 pellet-ChIP (no DNA-PKi) datasets after light exposure as indicated. NS=nonsignificant; **=p value<0.001, ***=p value < 0.0001. BLESS and LIG4 ChIP data[2] and BLISS and Mre11 ChIP (no DNA-PKi) data[3,4] are from previously published datasets.

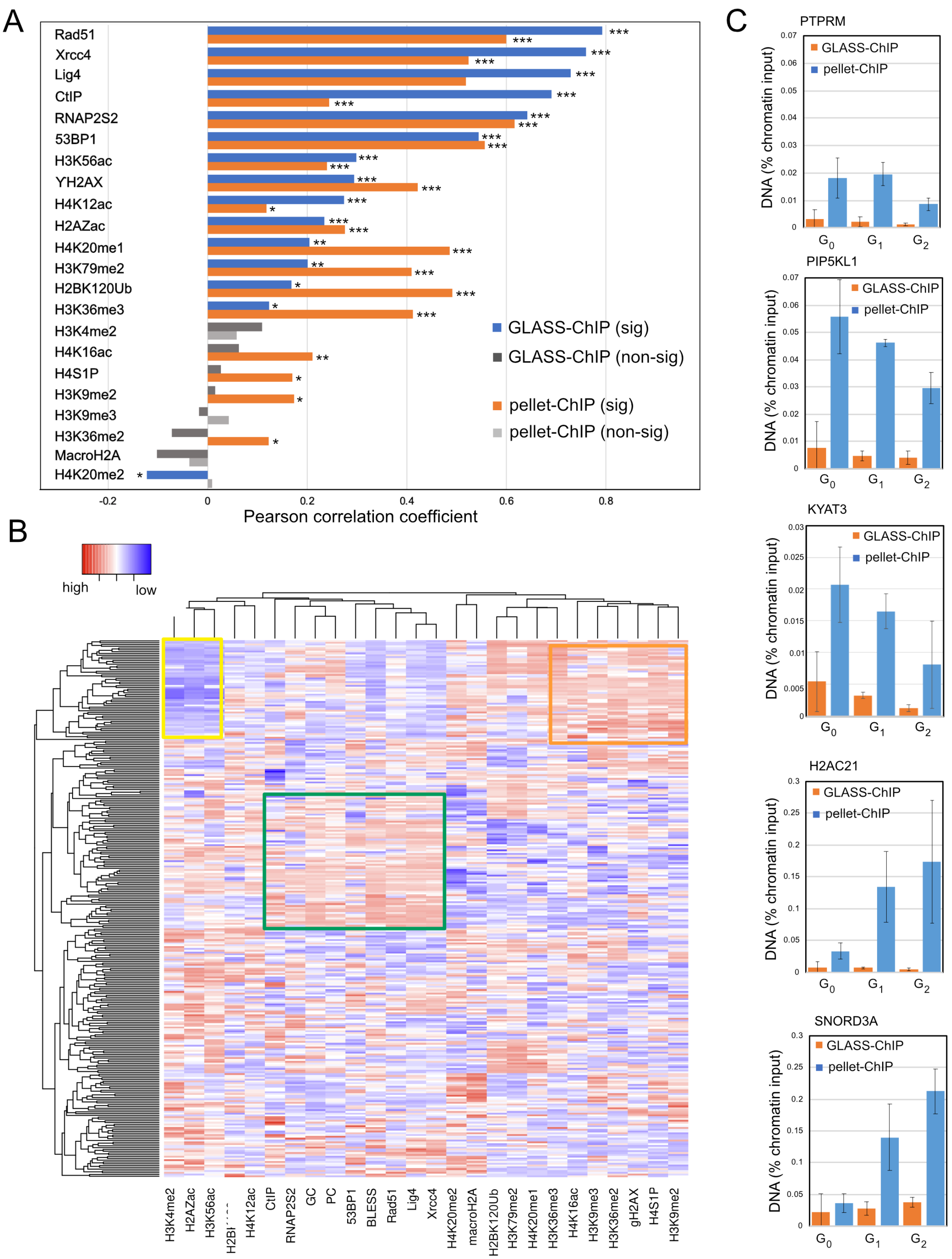
A
Rad51
Xrcc4
Lig4
CtIP
RNAP2S2
53BP1
H3K56ac
YH2AX
H4K12ac
H2AZac
H4K20me1
H3K79me2
H2BK120Ub
H3K36me3
H3K4me2
H4K16ac
H4S1P
H3K9me2
H3K9me3
H3K36me2
MacroH2A
H4K20me2
GLASS-ChIP (sig)
GLASS-ChIP (non-sig)
pellet-ChIP (sig)
pellet-ChIP (non-sig)
-0.2
0
0.2
0.4
0.6
0.8
Pearson correlation coefficient
B
high
low
H3K4me2
H2AZac
H3K56ac
H4K12ac
CtIP
RNAP2S2
GC
PC
53BP1
BLESS
Rad51
Lig4
Xrcc4
H4K20me2
macroH2A
H2BK120Ub
H3K79me2
H4K20me1
H3K36me3
H4K16ac
H3K9me3
H3K36me2
gH2AX
H4S1P
H3K9me2
C
PTPRM
PIP5KL1
KYAT3
H2AC21
SNORD3A
GLASS-ChIP
pellet-ChIP
DNA (% chromatin input)
G_0
G_1
G_2

Figure S4. DNA-PKcs released fragments and pellet-ChIP efficiency correlate with DSB markers. (A) Enrichment for DNA-PKcs in GLASS-ChIP and DNA-PKcs pellet-ChIP datasets in the absence of DNA-PKcs inhibition was compared with published data showing enrichment for DSB-associated binding factors and chromatin marks[2,5,6], generating correlation coefficients as shown. p values calculated using the linear regression t test; *, **, *** indicate $p<0.05$, 0.0005, and 0.00005, respectively. (B) Heat map showing unsupervised clustering of DNA-PKcs GLASS-ChIP (GC), pellet-ChIP (PC) and enrichment datasets showing DSB-associated binding factors and chromatin marks[2,5,6] using ranked enrichment data at the top 300 AsiSI sites. Boxes indicate clusters of breaks with similar patterns of DSB-associated marks (green), H3 and H4 modifications (orange), and low H3K56ac and associated marks (yellow). (C) Recovery of DNA-PKcs GLASS-ChIP and pellet-ChIP at 3 AsiSI sites (PTPRM, PIP5KL1, KYAT3) and two non-AsiSI genomic locations (H2AC21 and SNORD3A) by quantitative PCR. Error bars indicate standard deviation. Results are shown relative to chromatin input.

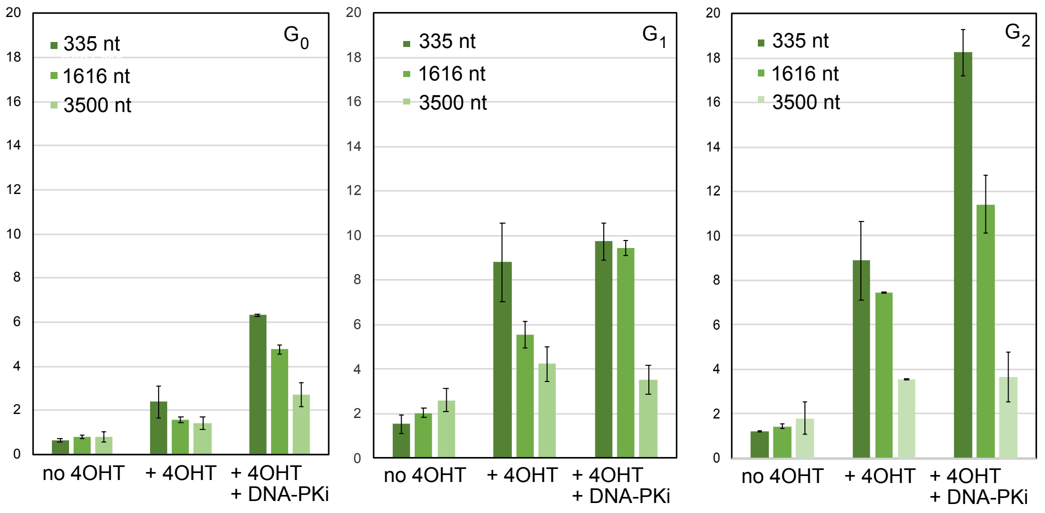

Figure S5. Resection at AsiSI breaks is observed in all cell cycle phases. Resection was monitored at various distances from the AsiSI site at the KYAT3 gene using a qPCR-based assay[7] in U2OS cells in different cell cycle phases with 4-OHT and DNA-PKi as indicated.

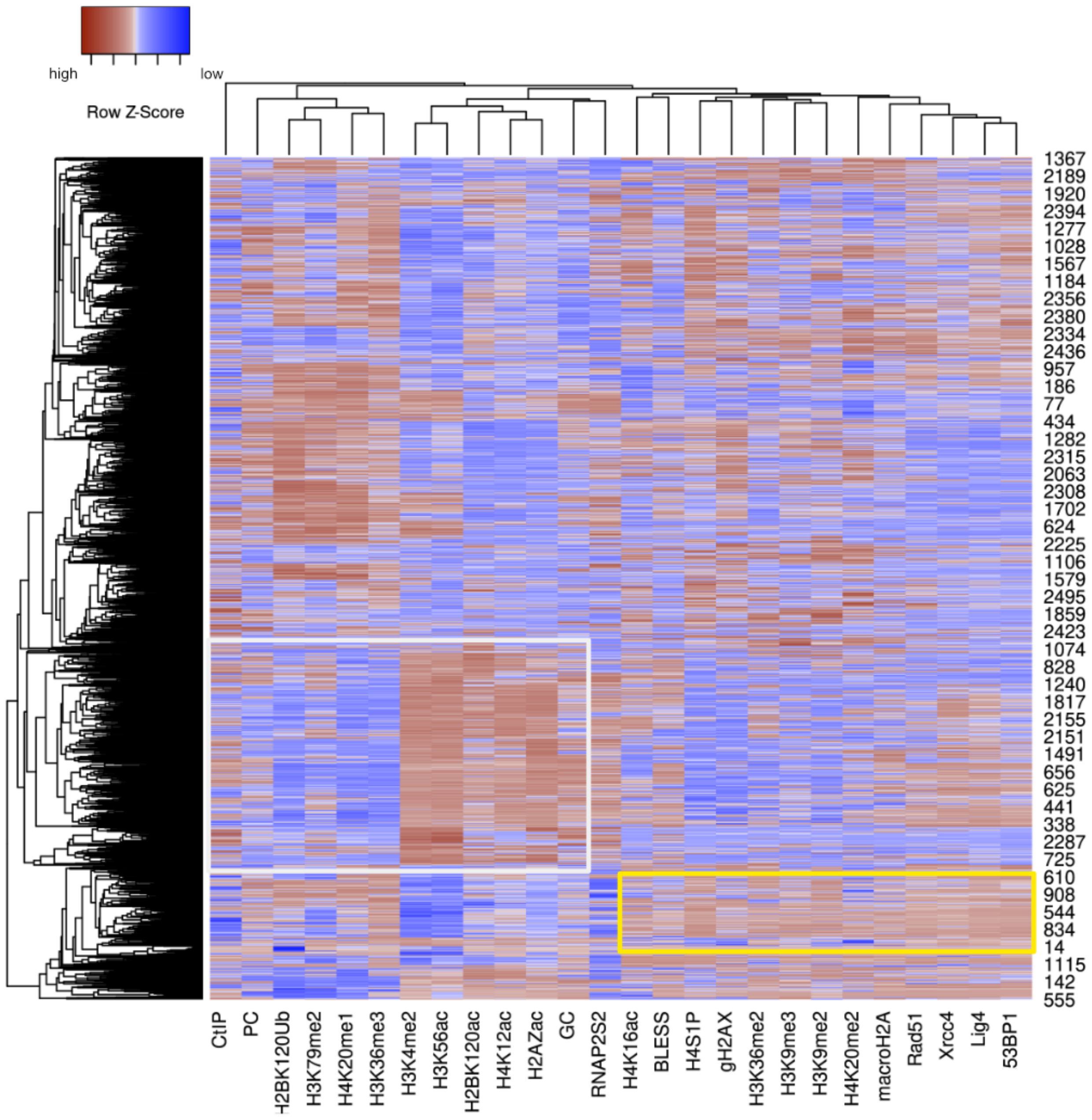

Figure S6. A subset of DNA-PKcs binding sites coincide with other DSB markers. The top 2500 binding sites from DNA-PKcs GLASS-ChIP were used to interrogate other DSB binding proteins and histone modifications from previous ChIP studies[1,2]. ChIP intensities for each factor/mark were used to generate rankings; these rankings were analyzed using unsupervised hierarchical clustering. Yellow box indicates one subset of sites where H4S1p, 53BP1, Rad51, Xrcc4, and Lig4 are enriched that contains many of the AsiSI sites; white indicates strongly anticorrelated marks present at a subset of GLASS-ChIP DNA-PKcs sites.

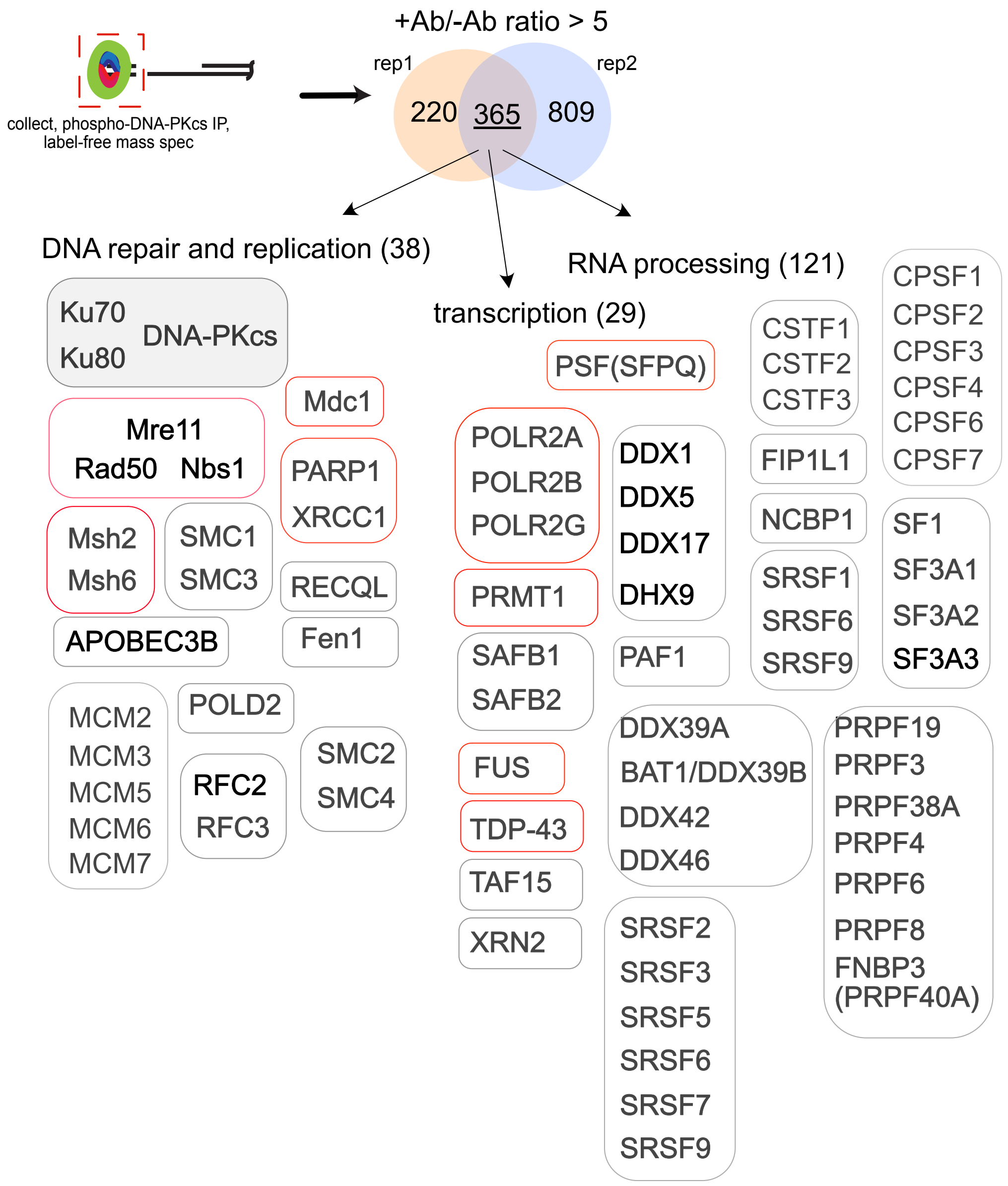

Figure S7. Mass Spectrometry identifies proteins associated with released DNA-PKcs GLASS-ChIP fragments. GLASS-ChIP was performed in two biological replicates, comparing -Ab to +Ab immunoprecipitations. Proteins were identified by label-free, quantitative mass spectrometry and targets with greater than 5-fold ratios between +Ab and -Ab were compared between the replicates as shown. Among the 365 targets are many DNA repair and replication factors as well as proteins involved in transcription and RNA processing. Factors previously known to associate with DNA-PK are outlined in red; not previously associated in grey. Complete list of factors in Supp. Data 1.

qPCR primers:		
Resection:		
3505	GAATCGGATGTATGCGACTGATC	top strand qPCR primer for AsiSI-DSB1(KYAT3) to amplify across BsrGI site
3506	TTCCAAAGTTATTCCAACCCGAT	bottom strand qPCR primer for AsiSI-DSB1(KYAT3) to amplify across BsrGI site
3508	TGAGGAGGTGACATTAGAACTCAGA	top strand qPCR primer for AsiSI-DSB1(KYAT3) to amplify across BsrGI site
3509	AGGACTCACTTACACGGCCTTT	bottom strand qPCR primer for AsiSI-DSB1(KYAT3) to amplify across BsrGI site
3511	TCCTAGCCAGATAATAATAGCTATACAAACA	top strand qPCR primer for AsiSI-DSB1(KYAT3) to amplify across BsrGI site
3512	TGAATAGACAGACAACAGATAAATGAGACA	bottom strand qPCR primer for AsiSI-DSB1(KYAT3) to amplify across BsrGI site
ChIP:		
7097	TCGGGGCCAGCGGCGTGTA	top strand qPCR primer 52bp upstream of AsiSI chr18 (PTPRM)
7128	CGCCAGCCCGCTCCC	bottom strand qPCR primer upstream at AsiSI site, use with TP7097
7105	GACTGCGGCTGCATCCAA	top strand qPCR primer towards ASiSI cut, 41bp upstream of AsiSI chr9 (PIP5KL1)
7120	CGCCAGCGCCTCCCGC	bottom strand qPCR primer at AsiSI site, use with TP7105
7254	GGCGGACCCCACCGTCAT	bottom strand qPCR primer for AsiSI site near KYAT3
3431	GATGTGGCCAGGGATTGG	top strand qPCR primer for AsiSI site near KYAT3
8090	GACACTTTGGTGGCTCTTAAAAGAGCCTTTG	top strand qPCR primer for H2AC21 non-AsiSI peak on Chr1
8091	CAAGAACAAGTAATTAAGAGGCTTGACACCATAC	bottom strand qPCR primer for H2AC21, use with TP8090
8094	CGAGGAAGAGAGGTAGCGTTTTCTCC	top strand qPCR primer for SNORD3A non-AsiSI peak on Chr17
8095	CAAGCAACGCCAGAAAGCCGGC	bottom strand qPCR primer for SNORD3A non-AsiSI peak on Chr17

Figure S8. qPCR primers used for resection and ChIP experiments.

1. Aymard, F. *et al.* Transcriptionally active chromatin recruits homologous recombination at DNA double-strand breaks. *Nat. Struct. Mol. Biol.* **21**, 366–374 (2014).
2. Clouaire, T. *et al.* Comprehensive Mapping of Histone Modifications at DNA Double-Strand Breaks Deciphers Repair Pathway Chromatin Signatures. *Molecular Cell* **72**, 250-262.e6 (2018).
3. Zou, R. S. *et al.* Massively parallel genomic perturbations with multi-target CRISPR interrogates Cas9 activity and DNA repair at endogenous sites. *Nat Cell Biol* **24**, 1433–1444 (2022).
4. Liu, Y. *et al.* Very fast CRISPR on demand. *Science* **368**, 1265–1269 (2020).
5. Cohen, S. *et al.* Senataxin resolves RNA:DNA hybrids forming at DNA double-strand breaks to prevent translocations. *Nat Commun* **9**, 533 (2018).
6. Aymard, F. *et al.* Transcriptionally active chromatin recruits homologous recombination at DNA double-strand breaks. *Nat. Struct. Mol. Biol.* **21**, 366–374 (2014).
7. Zhou, Y., Caron, P., Legube, G. & Paull, T. T. Quantitation of DNA double-strand break resection intermediates in human cells. *Nucleic acids research* **42**, e19 (2014).
