# Supplementary material for: Genome-wide analysis of DNA-PK-bound MRN cleavage products supports a sequential model of DSB repair pathway choice
Source: Nat Commun. 2023 Sep 16;14:5759. doi: 10.1038/s41467-023-41544-8 (PMC10505227; doi:10.1038/s41467-023-41544-8)
Supplement: Supplementary file 3 — Description of Additional Supplementary Files [file 41467_2023_41544_MOESM3_ESM.pdf]

Description of Additional Supplementary Files

File Name: Supplementary Data 1
Description: proteins identified by mass spectrometry from GLASS-ChIP immunoprecipitations.
