# Supplementary material for: Genome-wide analysis of DNA-PK-bound MRN cleavage products supports a sequential model of DSB repair pathway choice
Source: Nat Commun. 2023 Sep 16;14:5759. doi: 10.1038/s41467-023-41544-8 (PMC10505227; doi:10.1038/s41467-023-41544-8)
Supplement: Supplementary file 5 — Reporting Summary [file 41467_2023_41544_MOESM5_ESM.pdf]

nature portfolio

Corresponding author(s): Tanya Paull

Last updated by author(s): Jun 20, 2023

Reporting Summary

Nature Portfolio wishes to improve the reproducibility of the work that we publish. This form provides structure for consistency and transparency in reporting. For further information on Nature Portfolio policies, see our Editorial Policies and the Editorial Policy Checklist.

Statistics

For all statistical analyses, confirm that the following items are present in the figure legend, table legend, main text, or Methods section.

n/a	Confirmed	
☐	☒	The exact sample size (n) for each experimental group/condition, given as a discrete number and unit of measurement
☐	☒	A statement on whether measurements were taken from distinct samples or whether the same sample was measured repeatedly
☐	☒	The statistical test(s) used AND whether they are one- or two-sided<br>*Only common tests should be described solely by name; describe more complex techniques in the Methods section.*
☐	☒	A description of all covariates tested
☐	☒	A description of any assumptions or corrections, such as tests of normality and adjustment for multiple comparisons
☐	☒	A full description of the statistical parameters including central tendency (e.g. means) or other basic estimates (e.g. regression coefficient) AND variation (e.g. standard deviation) or associated estimates of uncertainty (e.g. confidence intervals)
☐	☒	For null hypothesis testing, the test statistic (e.g. F, t, r) with confidence intervals, effect sizes, degrees of freedom and P value noted<br>*Give P values as exact values whenever suitable.*
☒	☐	For Bayesian analysis, information on the choice of priors and Markov chain Monte Carlo settings
☒	☐	For hierarchical and complex designs, identification of the appropriate level for tests and full reporting of outcomes
☐	☒	Estimates of effect sizes (e.g. Cohen's d, Pearson's r), indicating how they were calculated

Our web collection on statistics for biologists contains articles on many of the points above.

Software and code

Policy information about availability of computer code

Data collection	All published algorithms and code used in the data collection are described and cited in the Methods section.
Data analysis	All published algorithms and code used in the analysis are described and cited in the Methods section.

For manuscripts utilizing custom algorithms or software that are central to the research but not yet described in published literature, software must be made available to editors and reviewers. We strongly encourage code deposition in a community repository (e.g. GitHub). See the Nature Portfolio guidelines for submitting code & software for further information.

Data

Policy information about availability of data

All manuscripts must include a data availability statement. This statement should provide the following information, where applicable:

- Accession codes, unique identifiers, or web links for publicly available datasets
- A description of any restrictions on data availability
- For clinical datasets or third party data, please ensure that the statement adheres to our policy

All raw sequencing files as well as processed data (bigwig files) have been deposited to GEO under record GSE218590. The following secure token has been created to allow review of while it remains in private status:ijobyiwaljqzdkr.

Research involving human participants, their data, or biological material

Policy information about studies with human participants or human data. See also policy information about sex, gender (identity/presentation), and sexual orientation and race, ethnicity and racism.

Reporting on sex and gender	N/A
Reporting on race, ethnicity, or other socially relevant groupings	N/A
Population characteristics	N/A
Recruitment	N/A
Ethics oversight	N/A

Note that full information on the approval of the study protocol must also be provided in the manuscript.

Field-specific reporting

Please select the one below that is the best fit for your research. If you are not sure, read the appropriate sections before making your selection.

☒ Life sciences ☐ Behavioural & social sciences ☐ Ecological, evolutionary & environmental sciences

For a reference copy of the document with all sections, see nature.com/documents/nr-reporting-summary-flat.pdf

Life sciences study design

All studies must disclose on these points even when the disclosure is negative.

Sample size	Sample sizes are noted in the figure kegends. For most sequencing datasets, two biological replicates were sequenced.
Data exclusions	No data exlusion
Replication	data shown in sequencing datasets were first obtained in preliminary form by quantitative PCR
Randomization	N/A
Blinding	N/A

Reporting for specific materials, systems and methods

We require information from authors about some types of materials, experimental systems and methods used in many studies. Here, indicate whether each material, system or method listed is relevant to your study. If you are not sure if a list item applies to your research, read the appropriate section before selecting a response.

Materials & experimental systems

n/a	Involved in the study	
☐	☒	Antibodies
☐	☒	Eukaryotic cell lines
☒	☐	Palaeontology and archaeology
☒	☐	Animals and other organisms
☒	☐	Clinical data
☒	☐	Dual use research of concern
☒	☐	Plants

Methods

n/a	Involved in the study	
☐	☒	ChIP-seq
☒	☐	Flow cytometry
☒	☐	MRI-based neuroimaging

Antibodies

Antibodies used	phospho2056 DNA-PKcs: Abcam ab124918<br>Mre11: Novus NB100-142<br>CtIP: Active Motif 61141

Validation | validation by western blot using DNAPKi or shRNA depletions to test for specificity

Eukaryotic cell lines

Policy information about cell lines and Sex and Gender in Research

Cell line source(s)	U2OS and HEK-293 cells: source ATCC
Authentication	ATCC
Mycoplasma contamination	ATCC
Commonly misidentified lines (See ICLAC register)	*Name any commonly misidentified cell lines used in the study and provide a rationale for their use.*

Plants

Seed stocks	*Report on the source of all seed stocks or other plant material used. If applicable, state the seed stock centre and catalogue number. If plant specimens were collected from the field, describe the collection location, date and sampling procedures.*
Novel plant genotypes	*Describe the methods by which all novel plant genotypes were produced. This includes those generated by transgenic approaches, gene editing, chemical/radiation-based mutagenesis and hybridization. For transgenic lines, describe the transformation method, the number of independent lines analyzed and the generation upon which experiments were performed. For gene-edited lines, describe the editor used, the endogenous sequence targeted for editing, the targeting guide RNA sequence (if applicable) and how the editor was applied.*
Authentication	*Describe any authentication procedures for each seed stock used or novel genotype generated. Describe any experiments used to assess the effect of a mutation and, where applicable, how potential secondary effects (e.g. second site T-DNA insertions, mosiacism, off-target gene editing) were examined.*

ChIP-seq

Data deposition

☒ Confirm that both raw and final processed data have been deposited in a public database such as GEO.

☒ Confirm that you have deposited or provided access to graph files (e.g. BED files) for the called peaks.

Data access links
May remain private before publication.

All raw sequencing files as well as processed data (bigwig and bed files) have been deposited to GEO under record GSE218590.

Files in database submission

ANGC3RE_S35_L001_R2_001.fastq.gz ANGC3RE_S35_L001_R1_001.fastq.gz ANGC3RE_S35_L002_R2_001.fastq.gz
ANGC3RE_S35_L002_R1_001.fastq.gz
ANUGC2RE_S34_L001_R2_001.fastq.gz ANUGC2RE_S34_L001_R1_001.fastq.gz ANUGC2RE_S34_L002_R2_001.fastq.gz
ANUGC2RE_S34_L002_R1_001.fastq.gz
no-4OHT_S8_L001_R1_001.fastq.gz no-4OHT_S8_L001_R2_001.fastq.gz no-4OHT_S8_L002_R2_001.fastq.gz
no-4OHT_S8_L002_R1_001.fastq.gz
ANUGC6RE_S38_L001_R2_001.fastq.gz ANUGC6RE_S38_L001_R1_001.fastq.gz ANUGC6RE_S38_L002_R2_001.fastq.gz
ANUGC6RE_S38_L002_R1_001.fastq.gz
ANUGC5R_S37_L001_R2_001.fastq.gz ANUGC5R_S37_L001_R1_001.fastq.gz ANUGC5R_S37_L002_R2_001.fastq.gz
ANUGC5R_S37_L002_R1_001.fastq.gz
plus-4OHT_S9_L001_R1_001.fastq.gz plus-4OHT_S9_L001_R2_001.fastq.gz plus-4OHT_S9_L002_R2_001.fastq.gz
plus-4OHT_S9_L002_R1_001.fastq.gz
AGC3_S23_L001_R2_001.fastq.gz AGC3_S23_L001_R1_001.fastq.gz AGC3_S23_L002_R2_001.fastq.gz
AGC3_S23_L002_R1_001.fastq.gz
TP8_S18_L001_R2_001.fastq.gz TP8_S18_L001_R1_001.fastq.gz TP8_S18_L002_R2_001.fastq.gz
TP8_S18_L002_R1_001.fastq.gz
AGC2_S22_L001_R2_001.fastq.gz AGC2_S22_L001_R1_001.fastq.gz AGC2_S22_L002_R2_001.fastq.gz
AGC2_S22_L002_R1_001.fastq.gz
TP7_S17_L001_R2_001.fastq.gz TP7_S17_L001_R1_001.fastq.gz TP7_S17_L002_R2_001.fastq.gz
TP7_S17_L002_R1_001.fastq.gz
AGC6RE_S26_L001_R2_001.fastq.gz AGC6RE_S26_L001_R1_001.fastq.gz AGC6RE_S26_L002_R1_001.fastq.gz
AGC6RE_S26_L002_R2_001.fastq.gz
TP10_S20_L001_R2_001.fastq.gz TP10_S20_L001_R1_001.fastq.gz TP10_S20_L002_R2_001.fastq.gz
TP10_S20_L002_R1_001.fastq.gz
AGC5R_S25_L001_R2_001.fastq.gz AGC5R_S25_L001_R1_001.fastq.gz AGC5R_S25_L002_R2_001.fastq.gz
AGC5R_S25_L002_R1_001.fastq.gz
TP9_S19_L001_R2_001.fastq.gz TP9_S19_L001_R1_001.fastq.gz TP9_S19_L002_R2_001.fastq.gz
TP9_S19_L002_R1_001.fastq.gz
ANUP9R_S41_L001_R2_001.fastq.gz ANUP9R_S41_L001_R1_001.fastq.gz ANUP9R_S41_L002_R2_001.fastq.gz
ANUP9R_S41_L002_R1_001.fastq.gz
ANUPC7_S39_L001_R2_001.fastq.gz ANUPC7_S39_L001_R1_001.fastq.gz ANUPC7_S39_L002_R2_001.fastq.gz

ANUPC7_S39_L002_R1_001.fastq.gz
ANUPC12R_S44_L001_R2_001.fastq.gz ANUPC12R_S44_L001_R1_001.fastq.gz ANUPC12R_S44_L002_R2_001.fastq.gz
ANUPC12R_S44_L002_R1_001.fastq.gz
NUPC10-1_S42_L001_R2_001.fastq.gz NUPC10-1_S42_L001_R1_001.fastq.gz NUPC10-1_S42_L002_R2_001.fastq.gz
NUPC10-1_S42_L002_R1_001.fastq.gz
AGC9_S29_L001_R2_001.fastq.gz AGC9_S29_L001_R1_001.fastq.gz AGC9_S29_L002_R2_001.fastq.gz
AGC9_S29_L002_R1_001.fastq.gz
AGC7_S27_L001_R2_001.fastq.gz AGC7_S27_L001_R1_001.fastq.gz AGC7_S27_L002_R2_001.fastq.gz
AGC7_S27_L002_R1_001.fastq.gz
APC12_S32_L001_R2_001.fastq.gz APC12_S32_L001_R1_001.fastq.gz APC12_S32_L002_R2_001.fastq.gz
APC12_S32_L002_R1_001.fastq.gz
AGC10_S30_L001_R2_001.fastq.gz AGC10_S30_L001_R1_001.fastq.gz AGC10_S30_L002_R2_001.fastq.gz
AGC10_S30_L002_R1_001.fastq.gz
TP2_S12_L001_R2_001.fastq.gz TP2_S12_L001_R1_001.fastq.gz TP2_S12_L002_R2_001.fastq.gz
TP2_S12_L002_R1_001.fastq.gz
TP1_S11_L001_R2_001.fastq.gz TP1_S11_L001_R1_001.fastq.gz TP1_S11_L002_R2_001.fastq.gz
TP1_S11_L002_R1_001.fastq.gz
TP4_S14_L001_R2_001.fastq.gz TP4_S14_L001_R1_001.fastq.gz TP4_S14_L002_R2_001.fastq.gz
TP4_S14_L002_R1_001.fastq.gz
TP3_S13_L001_R1_001.fastq.gz TP3_S13_L001_R2_001.fastq.gz TP3_S13_L002_R2_001.fastq.gz
TP3_S13_L002_R1_001.fastq.gz
TP6_S16_L001_R2_001.fastq.gz TP6_S16_L001_R1_001.fastq.gz TP6_S16_L002_R2_001.fastq.gz
TP6_S16_L002_R1_001.fastq.gz
TP5_S15_L001_R2_001.fastq.gz TP5_S15_L001_R1_001.fastq.gz TP5_S15_L002_R2_001.fastq.gz
TP5_S15_L002_R1_001.fastq.gz
TJ3_S1_L001_R1_001.fastq.gz TJ3_S1_L001_R2_001.fastq.gz TJ3_S1_L002_R2_001.fastq.gz TJ3_S1_L002_R1_001.fastq.gz
TJ4_S2_L001_R2_001.fastq.gz TJ4_S2_L001_R1_001.fastq.gz TJ4_S2_L002_R2_001.fastq.gz TJ4_S2_L002_R1_001.fastq.gz
TJ5_S3_L001_R2_001.fastq.gz TJ5_S3_L001_R1_001.fastq.gz TJ5_S3_L002_R2_001.fastq.gz TJ5_S3_L002_R1_001.fastq.gz
TJ6_S4_L001_R1_001.fastq.gz TJ6_S4_L001_R2_001.fastq.gz TJ6_S4_L002_R2_001.fastq.gz TJ6_S4_L002_R1_001.fastq.gz
TJ7_S5_L001_R2_001.fastq.gz TJ7_S5_L001_R1_001.fastq.gz TJ7_S5_L002_R1_001.fastq.gz TJ7_S5_L002_R2_001.fastq.gz
TJ8_S6_L001_R2_001.fastq.gz TJ8_S6_L001_R1_001.fastq.gz TJ8_S6_L002_R1_001.fastq.gz TJ8_S6_L002_R2_001.fastq.gz
TJ9_S1_L001_R1_001.fastq.gz TJ9_S1_L001_R2_001.fastq.gz
GC +DNAPKi no 4H +antibody, R1 and R2.bigwig

GC +DNAPKi plus 4H +antibody, R1 and R2.bigwig

GC no 4H +antibody R1 and R2.bigwig

GC plus 4H +antibody R1 and R2.bigwig

PC +DNAPKi no 4H +antibody.bigwig

PC +DNAPKi plus 4H +antibody.bigwig

PC no 4H +antibody.bigwig

PC plus 4H +antibody.bigwig

GC +DNAPKi plus 4H 1 hr only +antibody.bigwig

GC +DNAPKi plus 4H 1 hr only +PFM01 +antibody.bigwig

GC +DNAPKi plus 4H 1 hr only +PFM39 +antibody.bigwig

GC +DNA-PKi no light +antibody R1 and R2.bigwig

GC +DNA-PKi 15m post-light +antibody R1 and R2.bigwig

GC +DNA-PKi 60m post-light +antibody R1 and R2.bigwig

PC +DNA-PKi no light +antibody R1 and R2.bigwig

PC +DNA-PKi 15m post-light +antibody R1 and R2.bigwig

PC +DNA-PKi 60m post-light +antibody R1 and R2.bigwig

Mre11 +DNA-PKi no light +antibody R1 and R2.bigwig

Mre11 +DNA-PKi 15m post-light +antibody R1 and R2.bigwig

Mre11 +DNA-PKi 60m post-light +antibody R1 and R2.bigwig

GC +DNAPKi plus 4H 1 hr only +antibody.bigwig

GC +DNAPKi plus 4H 1 hr only +PFM01 +antibody.bigwig

GC +DNAPKi plus 4H 1 hr only +PFM39 +antibody.bigwig

CtIP GC, no 4-OHT, +antibody.bigwig

CtIP GC, plus 4-OHT, +antibody.bigwig

CtIP PC, no 4-OHT, +antibody.bigwig

CtIP PC, plus 4-OHT, +antibody.bigwig

DNAPK GC no 4OHT no DNAPKi, narrowpeak output with no Ab as control hg19.bed

DNAPK GC +4OHT no DNAPKi, narrowpeak output with no Ab as control hg19.bed

DNAPK GC no 4OHT +DNAPKi, narropeak output with no Ab as control hg19.bed

DNAPK GC +4OHT +DNAPKi, narrowpeak output with no Ab as control hg19.bed

DNAPK PC +4OHT no DNAPKi, narrowpeak output with input as control hg19.bed

DNAPK PC no 4OHT no DNAPKi, narrowpeak output with input as control hg19.bed

DNAPK PC no 4OHT +DNAPKi, narrowpeak output with input as control hg19.bed

DNAPK PC +4OHT +DNAPKi narrowpeak output with input as control hg19.bed

DNAPK GC no light +DNAPKi, narrowpeak output with input as control hg38.bed

DNAPK GC + light 15min +DNAPKi, narrowpeak output with input as control hg38.bed

DNAPK GC + light 60min +DNAPKi, narrowpeak output with input as control hg38.bed

DNAPK PC no light +DNAPKi, narrowpeak output with input as control hg38.bed

DNAPK PC + light 15min +DNAPKi, narrowpeak output with input as control hg38.bed

DNAPK PC + light 60min +DNAPKi, narrowpeak output with input as control hg38.bed
TJ10_S2_L001_R2_001.fastq.gz TJ10_S2_L001_R1_001.fastq.gz
TJ11_S3_L001_R2_001.fastq.gz TJ11_S3_L001_R1_001.fastq.gz
TJ12_S4_L001_R2_001.fastq.gz TJ12_S4_L001_R1_001.fastq.gz
TJ13_S5_L001_R2_001.fastq.gz TJ13_S5_L001_R1_001.fastq.gz
TJ14_S6_L001_R2_001.fastq.gz TJ14_S6_L001_R1_001.fastq.gz
MRE11_0m_R1_S1_R1_001.fastq.gz MRE11_0m_R1_S1_R2_001.fastq.gz
MRE11_0m_R2_S4_R1_001.fastq.gz MRE11_0m_R2_S4_R2_001.fastq.gz
MRE11_15m_R1_S2_R1_001.fastq.gz MRE11_15m_R1_S2_R2_001.fastq.gz
MRE11_15m_R2_S5_R1_001.fastq.gz MRE11_15m_R2_S5_R2_001.fastq.gz
MRE11_60m_R1_S3_R2_001.fastq.gz MRE11_60m_R1_S3_R1_001.fastq.gz
MRE11_60m_R2_S6_R1_001.fastq.gz MRE11_60m_R2_S6_R2_001.fastq.gz
TP2_S12_L001_R2_001.fastq.gz TP2_S12_L001_R1_001.fastq.gz TP2_S12_L002_R2_001.fastq.gz
TP2_S12_L002_R1_001.fastq.gz
TP1_S11_L001_R2_001.fastq.gz TP1_S11_L001_R1_001.fastq.gz TP1_S11_L002_R2_001.fastq.gz
TP1_S11_L002_R1_001.fastq.gz
TP4_S14_L001_R2_001.fastq.gz TP4_S14_L001_R1_001.fastq.gz TP4_S14_L002_R2_001.fastq.gz
TP4_S14_L002_R1_001.fastq.gz
TP3_S13_L001_R1_001.fastq.gz TP3_S13_L001_R2_001.fastq.gz TP3_S13_L002_R2_001.fastq.gz
TP3_S13_L002_R1_001.fastq.gz
TP6_S16_L001_R2_001.fastq.gz TP6_S16_L001_R1_001.fastq.gz TP6_S16_L002_R2_001.fastq.gz
TP6_S16_L002_R1_001.fastq.gz
TP5_S15_L001_R2_001.fastq.gz TP5_S15_L001_R1_001.fastq.gz TP5_S15_L002_R2_001.fastq.gz
TP5_S15_L002_R1_001.fastq.gz
HB29_S5_L001_R1_001.fastq.gz HB29_S5_L001_R2_001.fastq.gz
HB30_S6_L001_R1_001.fastq.gz HB30_S6_L001_R2_001.fastq.gz
HB35_S11_L001_R1_001.fastq.gz HB35_S11_L001_R2_001.fastq.gz
HB31_S7_L001_R1_001.fastq.gz HB31_S7_L001_R2_001.fastq.gz
HB32_S8_L001_R1_001.fastq.gz HB32_S8_L001_R2_001.fastq.gz
HB36_S12_L001_R1_001.fastq.gz HB36_S12_L001_R2_001.fastq.gz

HB25_S1_L001_R1_001.fastq.gz HB25_S1_L001_R2_001.fastq.gz
HB26_S2_L001_R1_001.fastq.gz HB26_S2_L001_R2_001.fastq.gz
HB33_S9_L001_R1_001.fastq.gz HB33_S9_L001_R2_001.fastq.gz
HB27_S3_L001_R1_001.fastq.gz HB27_S3_L001_R2_001.fastq.gz
HB28_S4_L001_R1_001.fastq.gz HB28_S4_L001_R2_001.fastq.gz
HB34_S10_L001_R1_001.fastq.gz HB34_S10_L001_R2_001.fastq.gz

Genome browser session
(e.g. UCSC)

N/A

Methodology

Replicates

ChIP-seq experiments were performed with biological replicates. ChIP-qPCR experiments were done with biological triplicate samples and technical replicates of the qPCR reactions

Sequencing depth

PE150, minimum of 15M reads per sample

Antibodies

phospho2056 DNA-PKcs: Abcam ab18192
Mre11: Novus NB100-142
CtIP: Active Motif 61141

Peak calling parameters

MACS2 peak calling was used. For GLASS-ChIP samples, no Ab was used as a control. For pellet ChIP samples, input samples were used as a control.

Data quality

For pellet ChIP, the effect of input was removed by using MACS bdgcmp with the ppois flag. The bedgraph output was further normalized for read depth within each experiment using multibigsummary with whole genome 10 kb bins to determine total signal for each sample, followed by normalization to 1M using bigwigcompare.

Software

fastqc, BWA-MEM, rmdup, MACS2 callpeak, MACS2 bdgcmp, multibigwigsummary, bigwigcompare
